# Behavioral and Metabolic Phenotype Indicate Personality in Zebrafish (*Danio rerio*)

**DOI:** 10.3389/fphys.2018.00653

**Published:** 2018-05-30

**Authors:** Mingzhe Yuan, Yan Chen, Yingying Huang, Weiqun Lu

**Affiliations:** ^1^National Demonstration Center for Experimental Fisheries Science Education, Shanghai Ocean University, Shanghai, China; ^2^The Key Laboratory of Exploration and Utilization of Aquatic Genetic Resources, Ministry of Education, Shanghai, China; ^3^International Research Center for Marine Biosciences at Shanghai Ocean University, Ministry of Science and Technology, Shanghai, China

**Keywords:** personality, behavior, aggression, environmental preference, metabolism, stress response

## Abstract

Consistency of individual differences of animal behavior and personality in reactions to various environmental stresses among their life stages could reflect basic divergences in coping style which may affect survival, social rank, and reproductive success in the wild. However, the physiological mechanisms determining personality remain poorly understood. In order to study whether behavior, metabolism and physiological stress responses relate to the personality, we employed post-stress recovery assays to separate zebrafish into two behavioral types (proactive and reactive). The results demonstrated consistent difference among personality, behavior and metabolism in which proactive individuals were more aggressive, had higher standard metabolic rates and showed lower shuttled frequencies between dark and light compartments than the reactive ones. The behavioral variations were also linked to divergent acute salinity stress responses: proactive individuals adopted a swift locomotion behavior in response to acute salinity challenge while reactive individuals remain unchanged. Our results provide useful insight into how personality acts on correlated traits and the importance of a holistic approach to understanding the mechanisms driving persistent inter-individual differences.

## Introduction

Fish individual behaviors are controlled by complex endogenous and exogenous factors. Individual differences can be represented as temper, behavioral syndrome (e.g., active vs. passive), coping styles and personality (Robinson, 2000; Réale et al., 2007; Carter et al., 2012; Rey et al., 2015). Stable phenotypic traits, including stress response, aggression, exploration and learning are generally divided into two groups: proactive and reactive, or bold and shy (Wilson, 1994; Koolhaas et al., 1999; Ariyomo, 2013). Proactive individuals are dominant in social hierarchies and have strong tendencies to take risks. In contrast, reactive individuals are subordinate in social hierarchies and tend to avoid risk. During acute stress, reactive individuals adopted a passive “freeze-hide” response by reducing their oxygen consumption rates (akin to shallow breathing) whereas proactive individuals adopted an active “fight-flight” response by increasing their rates of respiration (Buirski et al., 1978; Frost et al., 2013; Magno et al., 2015; Rupia et al., 2016). These behavioral differences may impact survival, social rank, and reproductive success, and these have been studied in a number of species, including fish (Øverli et al., 2004; Chen and Fernald, 2011; Tudorache et al., 2015). Here, we use the term “personality” to describe the consistent individual differences and “proactive and reactive” to describe the difference individual phenotype.

Previous studies have shown that not only behavioral traits, but also physiological traits vary between individuals when facing an environmental stress (Careau et al., 2008; Rupia et al., 2016). For instance, animals could perform a positive (fight-flight) or negative (freeze-hide) defense response during a predator encounter (Koolhaas et al., 1999). However, the link among personality, basic physiological processes and physiological responses under stress in fish still remains unclear. The incorporation of personality into physiological research gives a way to understand divergent stress responses, cognition, and the pace of life among individuals (Koolhaas et al., 1999; Burton et al., 2011). Studies have shown that personalities are associated with locomotion behavior and different cortisol responses after netting stress in both larvae and adult zebrafish (Tudorache et al., 2013, 2015). Metabolic rates are also suggested as one of the main drivers of personality variation in animals which can mediate energy consumption strategy (Biro and Stamps, 2010). For instance, consistent metabolic difference among individuals correlating with their personalities even across an environmental salinity gradient was demonstrated in Olive flounder (*Paralichthys olivaceus*) (Rupia et al., 2016). However, Polverino showed that body length, rather than rountine metabolic rate and body condition, correlated with activity and risk-taking in zebrafish (*Danio rerio*) (Polverino et al., 2016). Up to now, despite recent advances in the field, the integration of physiology and personality has not been determined for fish. Furthermore, the existence of different environmental stress responses between proactive and reactive individuals has not been fully addressed. However, this link is necessary to reinforce our mechanistic understanding of causal links among personality, behaviors, metabolism, and stress responses across contexts.

In this study, we examine the relationship between personality, behaviors, metabolism and acute stress responses in zebrafish (*D. rerio*), an important model organism for neuro-pharmacological, behavioral, and genetic research on stress (Steenbergen et al., 2011). Firstly, we use a post-stress recovery approach to identify the behavioral type. Then, behaviors and metabolism were tested to see if there were differences between two groups. Finally, we evaluate the acute stress response of individuals to a salinity challenge and compare whether there is a relationship between personality, behavior, metabolism, and stress response.

## Materials and methods

### Ethics statement

The experimental protocol was approved by the Animal Ethics committee of Shanghai Ocean University and abides by the Guidelines on Ethical Treatment of Experimental Animals established by the Ministry of Science and Technology, China.

### Animals and housing

The AB adult zebrafish (*Danio rerio*) used in the experiment was single batch offspring obtained from China Zebrafish Resource Center (CZRC). Fish were randomly selected and reared in an automatic recirculating water system (Haisheng, Shanghai, China) using a natural photoperiod (approximately 14 h light: 10 h dark) and a water temperature of 28 ± 0.5°C. Living artemia nauplii were used to feed the fish twice per day (8:00 a.m., 20:00 p.m.). To maintain the water quality, residual feeds were extracted from the tanks half an hour after feeding. In order to avoid the effect of sexual dimorphism, 23, 6-month-old female fish were used in this experiment. Before the experiment, fish were individually reared in one compartment of a standard zebrafish tank which separated into four compartments with transparent plastic board.

### Swimming parameters before and after exposure to stress

Between 9 and 11 a.m., 23 fish were individually transferred to cylindrical tanks (9 cm diameter, water depth 4 cm) without being exposed to air, then placed on light box of the zebrafish high-throughput behavior tester (ZEB6046, ViewPoint, France). After 15 min acclimation in the novel environment, the swimming parameters of fish were recorded for 15 min. Subsequently, a netting stressor was applied by catching fish with a net and lifting the net out of the water. The fish were suspended three times in air for 1 min, with two intervals of 30 s during which they were submerged in the water. Lastly, the fish were released again in the tanks after which we assessed the post-stress swimming parameters for 30 min (Tudorache et al., 2015). Post-stress period was divided into two periods, first 15 min (0–15 min), and second 15 min (16–30 min). The cumulative locomotion (CL) is the total locomotion that the fish swam during the recording time. Resting time (RT) is the total time that the fish did not swim during the recording time. Both parameters were measured using ViewPoint ZebraLab (ViewPoint, France). CL_0−15min_ and RT_0−15min_ values for post-stress were the swimming parameter of first 15 min after stress. CL_16−30min_ and RT_16−30min_ values for post-stress were the swimming parameter of second 15 min after stress. The absolute value of the difference of CL_0−15min_(CL_0−15min−Abs_) and CL_16−30min_ (CL_16−30min−Abs_), as well as RT_0−15min_(RT_0−15min−Abs_) and RT_16−30min_ (RT_16−30min−Abs_), between pre-stress and post-stress was the behavioral differences induced by netting stress. In general, a higher value of CL_Abs_ and RT_Abs_ indicates lower ability of fish to recover from a net stressor.

### Aggressive behavioral experiment

Aggressive behavioral experiments were conducted between 9 a.m. and 2 p.m. Before every experiment, the rectangular tank (measuring 30^*^12^*^16 cm, with a 16 ^*^ 12 cm mirror on one side) was sterilized with mild bleach solution. After triple wash with fresh water, the tank was filled to a height of 10 cm with aerated water, posited at 40 cm under a digital camera (uEye, IDS, Germany) on the uniform light board (~400 lux; measured at the water surface). An opaque cardboard was inserted at the middle of the tank before the initiation of the trial. The fish was transferred to the compartment without the mirror and acclimated for 10 min. Once acclimatized, the cardboard was lifted and the aggressive behaviors were recorded using the Shuttlesoft (Loligo systems, Denmark) for 480 s. Overall, three different measures were taken: overt aggression, restrained aggression and time spent close to the mirror (Balzarini et al., 2014). All aggressive behaviors were counted as events. For the overt aggression measure, the number of frontal attacks and biting attempts were calculated. For restrained aggression, the number of restrained behaviors was counted (Balzarini et al., 2014). The time spent by the fish in the 1 cm near the mirror was measured as the third event. Experiments were repeated three times with same fish. One-week recovery period was used between two experiments.

### Preference experiment

The preference experiments were conducted between 9 a.m. and 2 p.m. A rectangular tank (30^*^12^*^16 cm; 10 cm water depth), posited on the uniform light board (~400 lux; measured at the water surface), was split into two equal compartments, separated by an opaque cardboard. The surfaces of one compartment were white (light), while the others were black (dark). Both sides were not covered by a lid and the light under the tank was the only photosource used in this experiment (Manuel et al., 2014; Way et al., 2015). The tank was sterilized using mild bleach solution, with triple wash in freshwater before use. The recording devices and software were the same as the mirror test. The individual fish was transferred to the black compartment acclimating for 10 min and the behaviors were recorded for 480 s when the cardboard was lifted. Two parameters were measured: (1) the time spent in the dark compartment; (2) the shuttle frequencies between dark and light compartments. Experiments were repeated three times with same fish. One-week recovery period was used between two experiments.

### Respiration test

An automated intermittent-flow respirometry (AutoRespTM, Loligo Systems, Denmark) to measure an individual's oxygen consumption rate (MO_2_; mgO_2_ g^−1^ h^−1^), which were used to estimate the upper and lower bounds of a fish's capacity to metabolize oxygen: the maximum metabolic rate (MMR, estimated as MO_2_, max after exhaustive exercise) and standard metabolic rate (SMR, estimated as MO_2_, min in resting fish). MMR is related to an animal's lifestyle both within and among species and sets the ceiling for aerobic metabolic performance (Binning et al., 2014, 2015; Norin and Clark, 2016). High and low MMR are associated with more athletic and sedentary lifestyles, respectively (Norin and Clark, 2016). On the other hand, SMR represents the energetic cost of self-maintenance with high SMR requiring more resources to sustain basic life functions than low SMR individuals or species(Burton et al., 2011; Chabot et al., 2016a,b; Metcalfe et al., 2016). The difference between these two measures, the absolute aerobic scope (AAS), represents the metabolic confines within which aerobic activities must take place and is linked to whole organism performance and fitness (Metcalfe et al., 2016). Respirometry trials were carried out in horizontal mini chambers (24 mm diameter) designed by Loligo Systems. All devices were sterilized using mild bleach solution and washed completely before using. Chambers were submerged in 8 L aerated water bath, using a temperature analyzer and regulator system (TMP-REG, Loligo Systems, Denmark) to maintain the water temperature at 28 ± 0.5°C. Oxygen levels in the respirometer were recorded using a galvanic oxygen electrode with the AutoResp^TM^ 4 LDAQ software (Loligo Systems, Denmark). All respirometry experiments were conducted at 9 am according to an air exposure protocol used as Rupia described (Rupia et al., 2016) to obtain MMR, followed by over 24 h of recovery and continuous monitoring of MO_2_ to obtain SMR. Ten-minutes loops were used with a 240-s flush, 60-s wait, and 300-s measure cycle. The body parameters (body length, width, height, and wet weight) of each individual were inputted into the respirometry software to correct the metabolic rate. Three loops were run without a fish to measure initial and final background rates of respiration due to bacterial load in the test chamber in order to minimum the error. We estimated standard metabolic rate (SMR) as the average of the10% lowest MO_2_ measurements during the trial period (Jordan et al., 2001; Chabot et al., 2016b). AAS was calculated as MMR—SMR. The R_2_ of the data that we used was higher than 0.9 (Svendsen et al., 2016).

### Acute salinity challenge experiment

Between 9 and 11 a.m., 23 fish were transferred to cylindrical tanks (9 cm diameter, water depth 4 cm), individually, then placed on light box of the zebrafish high-throughput behavior tester (ZEB6046, ViewPoint, France). After 15 min acclimation in the novel environment, the swimming parameters of fish were recorded for 15 min. Subsequently, fish were switched to salinity treatment (15 ppt) and recorded for 15 min. Fish were put back to the home tank after the acute salinity challenge. Seven days later, the acute salinity challenge experiments were induced again. The cumulative locomotion (CL) and resting time (RT) were measured using ViewPoint ZebraLab (ViewPoint, France). CL_AVG_ and RT_AVG_ values for freshwater and salinity treatment (15 ppt) were the averages over corresponding experimental periods of 15 min. The absolute value of the difference of CL_AVG_, as well as RT_AVG_, between freshwater and salinity treatment were the stress behavioral differences (CL_AVG−Abs_, RT_AVG−Abs_).

### Statistical analysis

Recovery parameters were clustered by hierarchical clustering analyses (PRIMER6, PRIMER-E Ltd; Ward's linkage method) to assign individuals into groups reflecting behavioral types, which categorized fish as proactive and reactive (Rupia et al., 2016). Data were tested for normality by the Shapiro-Wilk's test and homogeneity of variance by Levene's test, and expressed as means ± S.E.M. Significant differences between groups were analyzed by Student's *t*-test and the data accorded with the request of Student's *t*-test. Results were considered significantly different when *p* < 0.05. The analyses were conducted using a computer program, GraphPad Prism 5.0 (San Diego, CA, USA). A principal component analysis (PCA) was used to create composite behavioral scores for each individual based on seven measures of their performance during the behavioral assays (XLSTAT®2014) (Wang et al., 2015). A biplot was constructed showing both the measured variables and the observations. The relationship between composite behavioral scores, which were factor scores of PC1, and metabolism were tested with linear regression by GraphPad Prism 5.0. The repeatability of acute salinity challenge behaviors were estimated at Day 1 and Day 8 using two product-moment correlations (Perason's r) by GraphPad Prism 5.0 (Rupia et al., 2016).

## Results

### Swimming parameters before and after exposure to stress

**Figure 1:**
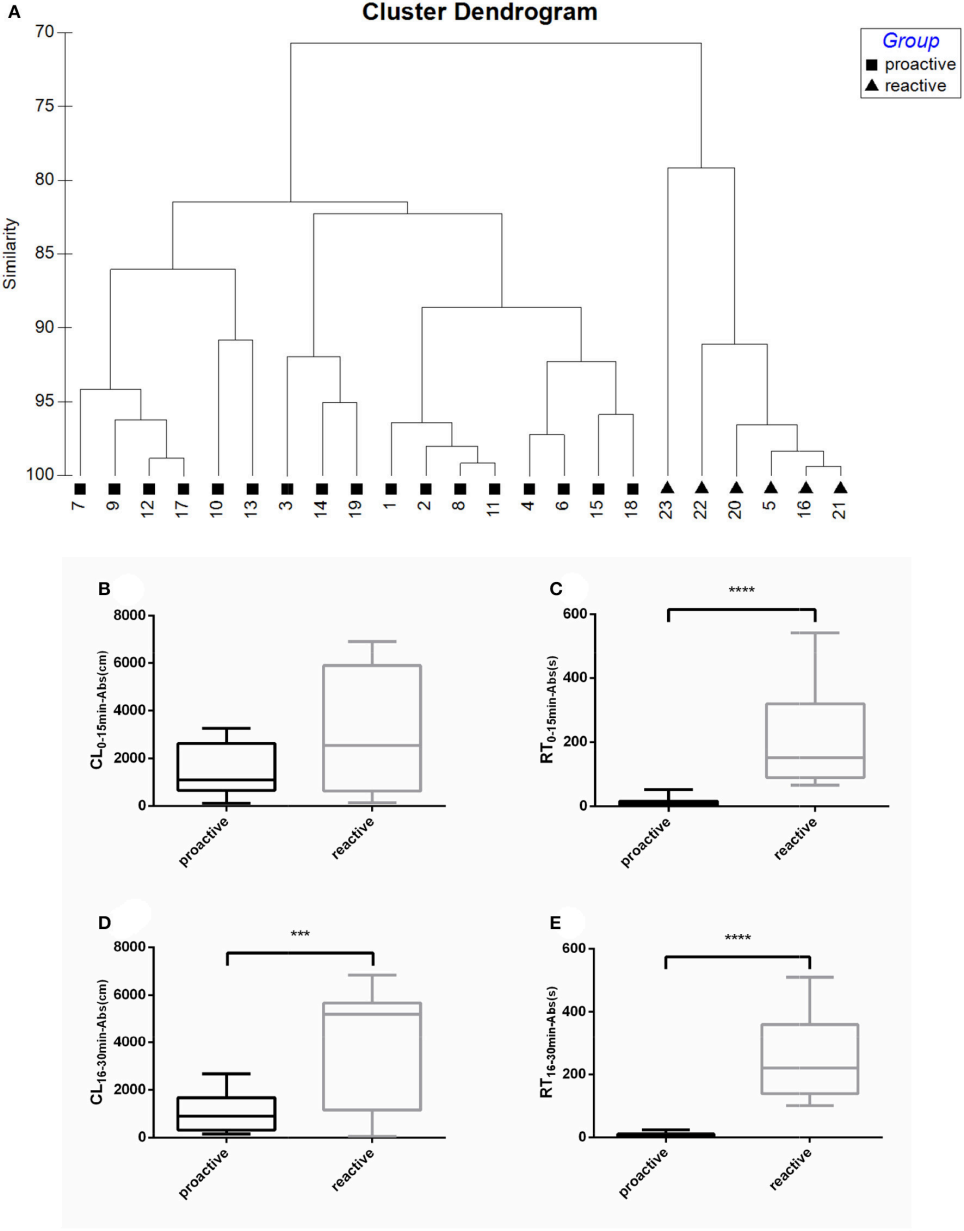
Swimming parameters were analyzed before and after exposure to stress. **(A)** Cluster dendrogram showing the proactive (■) and reactive (▴) groups identified by hierarchical clustering analysis (Ward's linkage method) on recovery parameters (CL_16−30min−Abs_, RT_16−30min−Abs_). Fish ID and group are presented at the end of each branch. **(B)** CL_0−15min−Abs_ between proactive and reactive group. **(C)** RT_0−15min−Abs_ between proactive and reactive group. **(D)** CL_16−30min−Abs_ between proactive and reactive group. **(E)** RT_0−15min−Abs_ between proactive and reactive group. Significantly different from the proactive (*n* = 17) and reactive (*n* = 6) group at *P* < 0.001 and 0.0001, by Student's *t*-test^***^, ^****^.

Fish were clustered into two groups based on their recovery parameters (CL_16−30min−Abs_, RT_16−30min−Abs_), suggesting different post-stress reactions between “Proactive” and “Reactive” individuals (Figure 1A). No difference of CL_0−15min−Abs_ was observed between two groups in the first 15 min following netting stress (Figure 1B). Proactive individuals are defined as the fish which have stronger abilities to recover from the any stress compared with the reactive one. The CL_16−30min−Abs_, RT_0−15min−Abs_, and RT_16−30min−Abs_ of proactive individuals showed significantly lower than reactive individuals (*P* < 0.001; Figures 1C–E).

### Aggressive behavioral experiment

**Figure 2:**
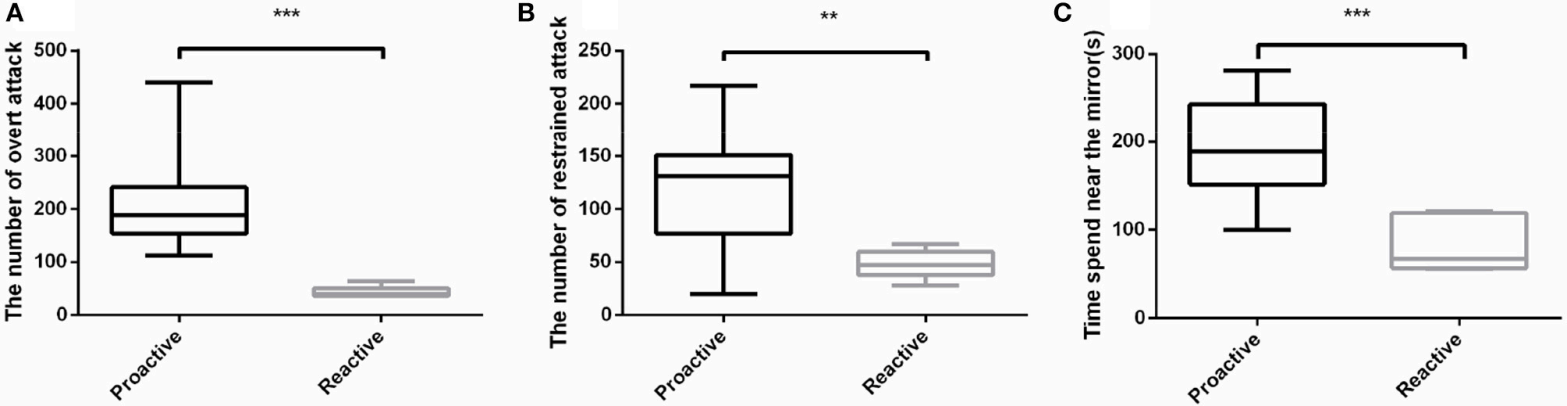
Aggressive behavioral experiments were conducted between two types of fish. **(A)**The number of overt attack. **(B)** The number of restrained attack. **(C)** Time spend near the mirror. Significantly different from the proactive (*n* = 17) and reactive (*n* = 6) group at *P* < 0.01 and 0.001, by Student's *t*-test^**^, ^***^.

In order to evaluate the aggressive behavior between proactive and reactive individuals, Mirror tests were conducted. Three parameters were measured to assess the aggressive behaviors between the two types of fish. Among the three aspects of the aggressive behaviors, proactive individuals not only performed a dramatically higher frequency of both overt (*P* < 0.001; Figure 2A) and restrained (*P* < 0.01; Figure 2B) attacks than the reactive ones, but also spent more time in the 1 cm near the mirror (*P* < 0.001; Figure 2C). However, only overt attack showed strict dimorphism between two types in which all proactive individuals performed higher overt attack level. Overall, proactive individuals showed more aggression than reactive one (Videos 1, 2).

### Preference experiment

**Figure 3:**
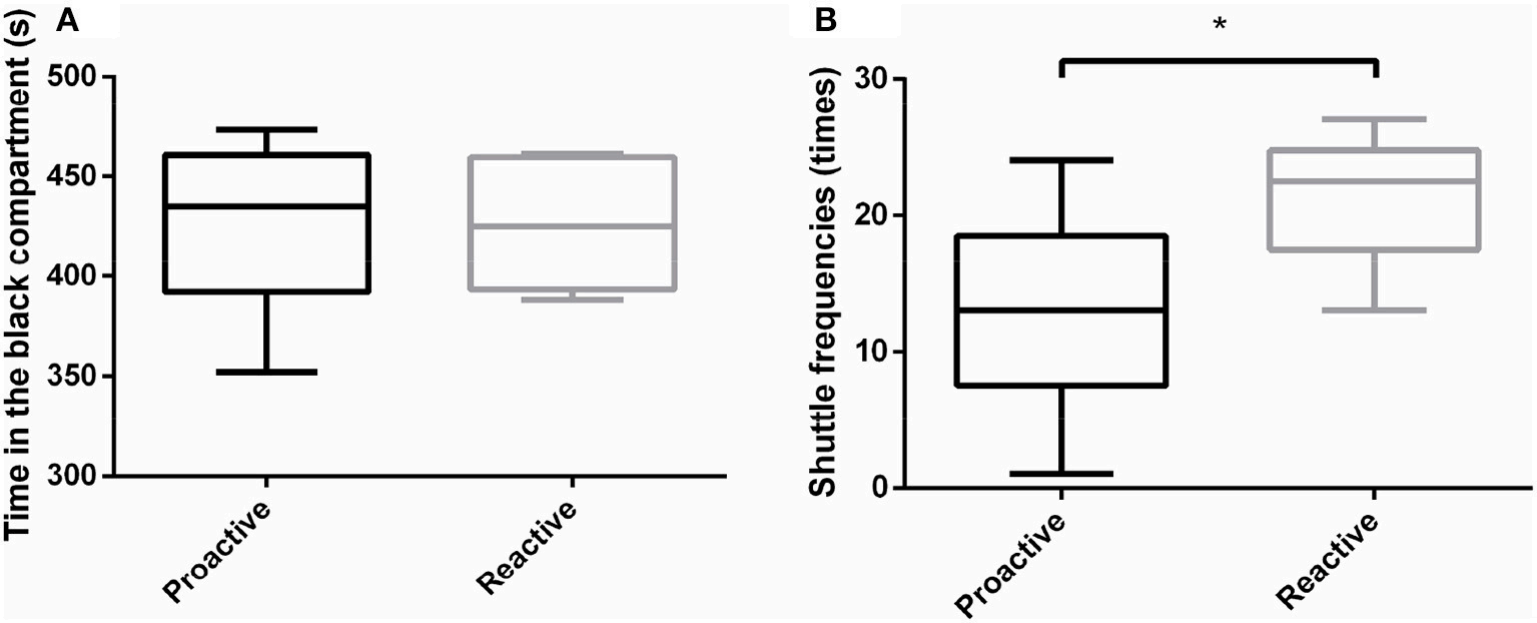
Environmental preference experiments were conducted between two types of fish. **(A)**The time of different individuals spent in the black compartment. **(B)** The shuttle frequencies between black and white compartments. Significantly different from the proactive (*n* = 17) and reactive (*n* = 6) group at *P* < 0.05, by Student's *t*-test^*^.

In order to analyze the environmental preference between two groups, black-white box experiments were carried out. The results revealed that both proactive and reactive individuals spent more time in dark compartment and there were no significant differences in the total time in the dark compartment between the two types of fish (Figure 3A). However, reactive fish showed significantly higher shuttle frequencies between dark and light compartments, compared with proactive fish (*P* < 0.05; Figure 3B).

### Links between personality and metabolism

**Figure 4:**
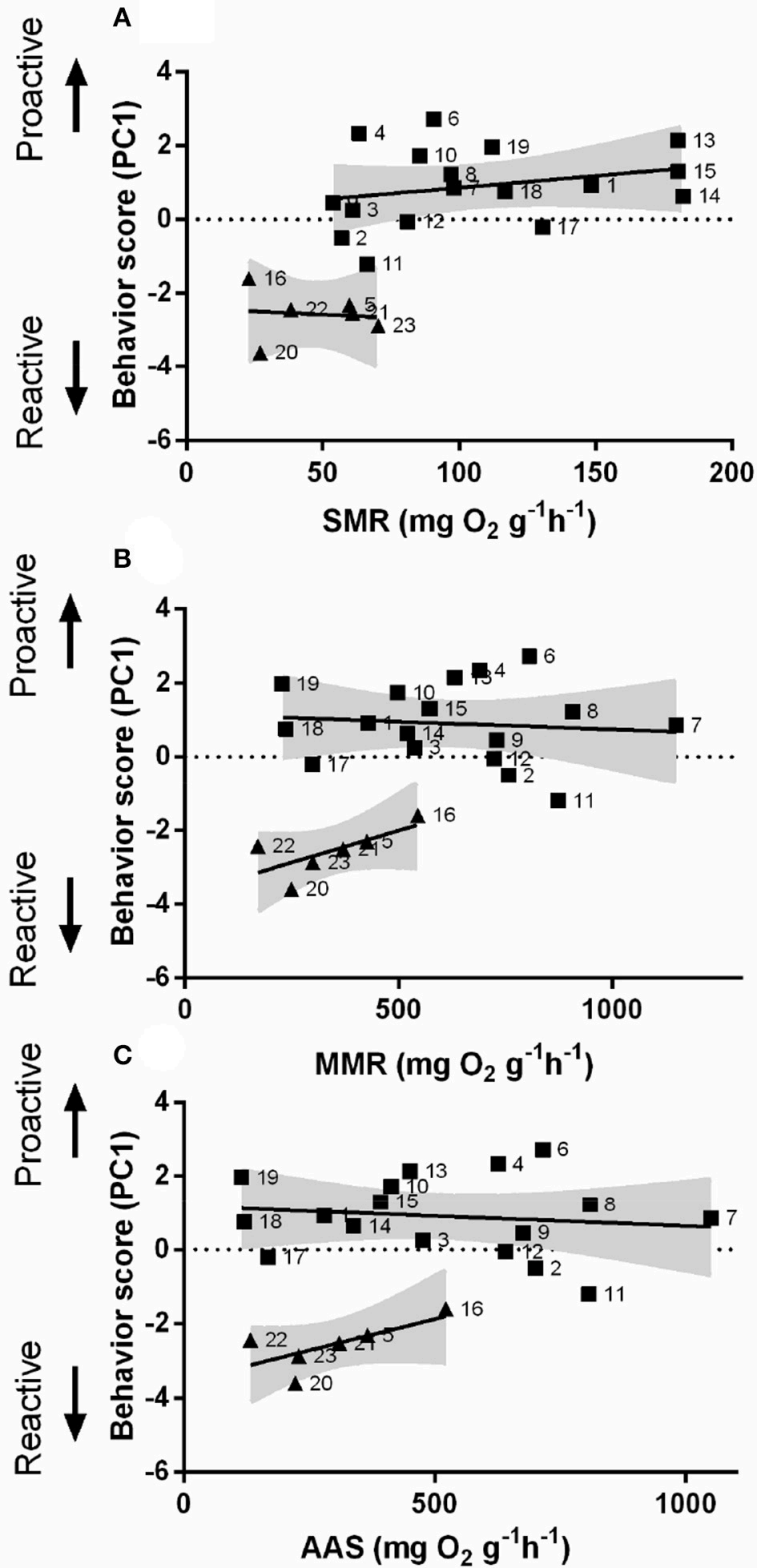
Composite behavioral score as a function of metabolism. The relationship between behavioral score (from reactive to proactive) and **(A)** standard metabolic rate (SMR), **(B)** maximum metabolic rate (MMR), and **(C)** absolute aerobic scope (AAS) in mg O_2_ g^−1^ h^−1^ for zebrafish. Square dots represent individuals with high composite behavioral score (proactive) (*n* = 17); Triangular dots represent individuals with a low composite behavioral score (reactive) (*n* = 6); the gray area represents the 95% confidence interval.

In order to understand the link between personality and non-stress metabolic performance, an automated intermittent-flow respirometry device was used. Behavior scores (PC1) were obtained from PCA (Figure S1 and Table S1). SMR differed significantly between proactive and reactive individuals [Linear regression; *F*_(1, 20)_ = 31.34, *P* < 0.01, Figure 4A]. Proactive and reactive fish also exhibited significant differences in MMR [Linear regression; *F*_(1, 20)_ = 45.92, *P* < 0.05, Figure 4B] but not in AAS [Linear regression; *F*_(1, 20)_ = 39.89, *P* > 0.05, Figure 4C].

### Acute salinity challenge responses

**Figure 5:**
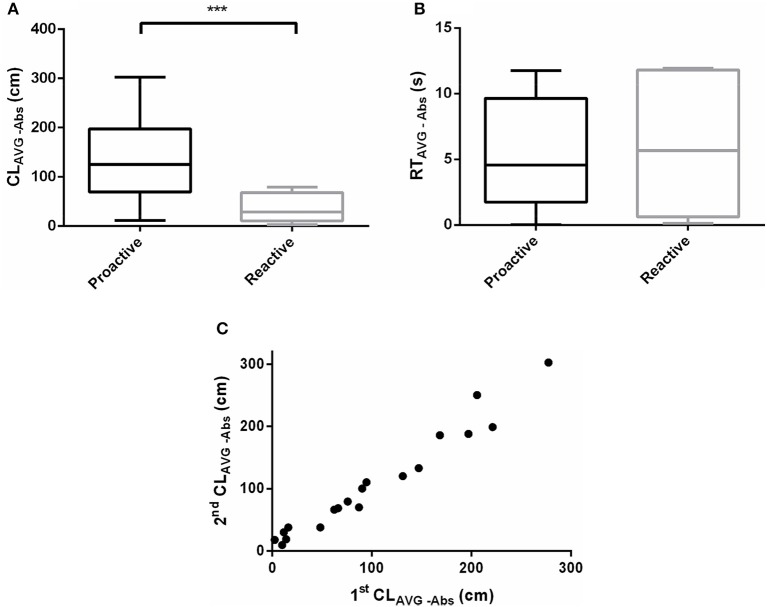
Swimming parameters were analyzed before and after exposure to acute salinity challenge. **(A)** CL_AVG−Abs_ between proactive and reactive group, **(B)** RT_AVG−Abs_ between proactive and reactive group, and **(C)** correlations between stress behavior (CL_AVG−Abs_, *r* = 0.9793) of individual across time of Day 1 (1st CL_AVG−Abs_) and Day 8 (2nd CL_AVG−Abs_). Significantly different from the proactive (*n* = 17) and reactive (*n* = 6) group at *P* < 0.001, by Student's *t*-test^***^.

The effect of acute salinity challenge responses differed between proactive and reactive fish. The CL_AVG−Abs_ of proactive individuals was significantly higher than the reactive one after acute salinity treatment (*P* < 0.001, Figure 5A). Nevertheless, RT_AVG−Abs_ didn't show dimorphism between the two groups (*P* > 0.05, Figure 5B). The CL_AVG−Abs_ (Pearson's correlation; slope = 1.027, d.f. = 17, *P* < 0.0001, *r* = 0.9793) were highly repeatable across time (Figure 5C, Videos 3–6).

## Discussion

In this study, two periods were recorded after netting stress, first 15 min and second 15 min. The results showed that two types of fish would perform same stress behavior within first 15 min after netting stress. Stress behavior showed divergent between two types of fish in second 15 min. According to the second 15 min swimming parameter of post-stress deviate from pre-stress (CL_16−30min−Abs_ and RT_16−30min−Abs_), fish were divided into two groups, proactive, and reactive, which showed visual difference in post-stress recovery.

Previous studies had used diversiform ways to divide conspecific animal into groups in order to evaluate individual differences between fish (Rupia et al., 2016), mammals (Oortmerssen and Busser, 1989), and birds (Favati et al., 2014). Tudorache and colleagues divided both adult and larvae zebrafish into two types according to the latency time of fish emerged from a dark environment into a light one. They found that the early to emerge individuals recovered quicker than late to emerge one after a net stress, not only the swimming behavior, but also the whole-body cortisol (Tudorache et al., 2013, 2015). Other researches also used anti-predator (Rupia et al., 2016), food seeking (Ruizgomez et al., 2011), and social hierarchy behaviors (Zajitschek et al., 2017) as the basis of classification to distinguish conspecific fish into groups, typically called “bold and shy” or “proactive and reactive.” In this study, we separated trial fish by recovery parameters of CL_Abs_, RT_Abs_, which presented the ability of fish recovering from a net stressor, as proactive (recovered quicker) and reactive (recovered slower) individuals.

It has been well accepted that various behaviors of conspecific animals reflect their different environmental coping strategies, or called “personality.” In order to split the large population into groups in a relatively short time, mirror test and black-white box experiment were designed to explore whether there is a repeatable relevance among personality, aggression and environmental preference (Balzarini et al., 2014; Manuel et al., 2014; Way et al., 2015).

Aggression is a psychological construct that is commonly used to classify animal behavior. Although aggression is complex and difficult to accurately quantify, we exploited the concept of a widely-used mirror test, modified for the use with zebrafish. Previous studies revealed that proactive individuals demonstrated higher aggressive level than the reactive one in many species (Koolhaas et al., 2007; Thomson et al., 2011; Way et al., 2015). Our results showed more details about the aggression between the two behavioral types in which the proactive individuals stayed longer near the mirror and performed more offensive attacks, not only overt but also restrained attacks, than the reactive one. Our results indicated the proactive individuals were more aggressive than the reactive ones.

Black-white box experiments are widely used to assess of cognitive, exploring, learning ability and environmental preference (Manuel et al., 2014; Naderi et al., 2016). Previous studies found that the adult zebrafish displayed light-avoidance behavior, which was anxiety-related (Bai et al., 2016). In our experiment, we used black-white box test to identify whether there is any environmental preference (especially in light preference) differences between two groups. Light avoidance was observed in our experimental fish with no difference between two groups. However, it was more frequent for reactive fish shuttled back and forth between the two compartments than the proactive one, which might indicate that the reactive fish were more anxious in a novel environment. These results broadly reflect the willingness of proactive individuals to take risks and vie for the social status. It had been suggested that the phenotypic differences have a genetic component due to the disruptive selection for extremes of the phenotypic variation (Koolhaas et al., 1999). However, whether there is strong genetic difference between the proactive and reactive zebrafish has not been found yet, but at least, there should be some physiological process which administrates the phenotype.

Previous studies have shown various physiological responses of individuals exposed to a stressful environment (Frost et al., 2007). However, whether there is any base-line difference within the individual-level in zebrafish remained unknown. Standard metabolism indicates the lowest oxygen consumption of aerobic metabolism in the non-moving state of an organism under certain environmental conditions, which can be used as an indicator to assess the level of fish base activity and energy consumption(Farrell et al., 2003; Steinhausen et al., 2008; Andersson and Hoglund, 2012). Some studies have suggested that metabolic performance varies across personality and environment which means that the personalities are flexible in response to biotic challenges (Frost et al., 2007). However, Rupia and colleagues found that the individual-level measures of personality and metabolic performance remained constant between both adaptable environment and contexts (Rupia et al., 2016). In our studies, PCA was used to reduce the dimension of complex behavioral performance, in order to explore the correlation between the behavioral performance and metabolism. Three metabolic parameters (e.g., SMR, MMR, AAS) were analyzed. Our results showed that PC1 were positively relevant to the proactive individuals, and proactive individuals had higher SMR and MMR. And the separation between types became clearer with measures of standard metabolic rate, suggesting that the grouping of two behavioral types had various baseline of energy consumption. This dimorphism were also discovered in other species, such as catfish (McKenzie et al., 2015) and flounder (Rupia et al., 2016). Nevertheless, AAS didn't appear to follow the same trend.

As many studies have pointed that different personalities such as proactive and reactive show divergent behavioral and physiological responses when facing environmental stress (Øverli and Winberg, 2005; Careau et al., 2008). The presence of individual differences would enhance the overall environmental adaptability of the population by reducing the overlap of the niche (Bertolo et al., 2011). The acute stress responses are usually attributed to the variation in the degree of hypothalamus-pituitary-interrenal (HPI) activity (Øverli et al., 2007). Tudorache and colleagues found that individuals would perform different secretion pattern of whole-body cortisol concentrations after a net stress, in which the cortisol level of proactive individuals showed significant increase (Tudorache et al., 2015). Studies have shown the role of behavior in osmotic challenges (Wolcott and Wolcott, 2001). Behaviors include evasion of stressful habitats, selection among differing microenvironments, changing body characteristics that affect salt/water uptake/loss, manipulating fluids differing in osmolytes, and modification of osmotic microenvironments (Wolcott and Wolcott, 2001). Here, we showed that the behavioral response to an acute salinity challenge also differ between proactive and reactive individuals. After 15 min acute salinity challenge, proactive individuals rapidly reduced cumulative locomotion while the reactive ones remain unchanged. These divergent behavioral responses suggested that proactive individuals had a swift response to the salinity changes whereas reactive individuals were negative and remained immobile when facing environmental stress. This may cause the reactive individuals more vulnerable.

## Conclusion

The present study shows that different personalities are present in female adult zebrafish, and behavioral and metabolic characteristics of coping styles are related. The data on post-stress recovery separated fish into proactive and reactive groups. Furthermore, the proactive individuals are more aggressive; higher SMR and MMR; less shuttled between black-white box; quick response to acute salinity challenge than reactive individuals. Consequently, our results showed the differences among behavioral, metabolic profiles and the acute salinity challenge responses in two behavior groups, which provide useful insight into how selection acts on correlated traits and the importance of a holistic approach to understanding the mechanisms driving persistent inter-individual differences.

## Author contributions

WL and MY designed experiments and wrote the manuscript. YC, YH, and MY carried out experiments. MY analyzed experimental results.

### Conflict of interest statement

The authors declare that the research was conducted in the absence of any commercial or financial relationships that could be construed as a potential conflict of interest.
