# Supplementary material for: Behavioral and Metabolic Phenotype Indicate Personality in Zebrafish (Danio rerio)
Source: Front Physiol. 2018 May 30;9:653. doi: 10.3389/fphys.2018.00653 (PMC5988878; doi:10.3389/fphys.2018.00653)
Supplement: Supplementary file 7 [file Image_1.pdf]

Online supporting Information

Behavioral and metabolic phenotype indicate personality in zebrafish (*Danio rerio*)

Mingzhe Yuan[1,2†], Yan Chen[1†], Yingying Huang[1†] and Weiqun Lu[1,2,3*]

1 National Demonstration Center for Experimental Fisheries Science Education, Shanghai Ocean University, Shanghai, 201306, China

2 The Key Laboratory of Exploration and Utilization of Aquatic Genetic Resources, Ministry of Education, Shanghai, 201306, China

3 International Research Center for Marine Biosciences at Shanghai Ocean University, Ministry of Science and Technology, China

† Joint first authors

*Corresponding author: wqlv@shou.edu.cn

Supplemental Data

Fig. S1 Scatter diagram of the principal component analysis (PCA) for six behavioral measures of fish maintained in: (A) Time spent in 1 cm near the mirror, (B) The number of restrained attract, (C) The number of overt attacks, (D) shuttled frequencies, (E) $CL_{16\text{-}30min\text{–}Abs}$, (F) $RT_{16\text{-}30min\text{ -}Abs}$. See the methods section for details.

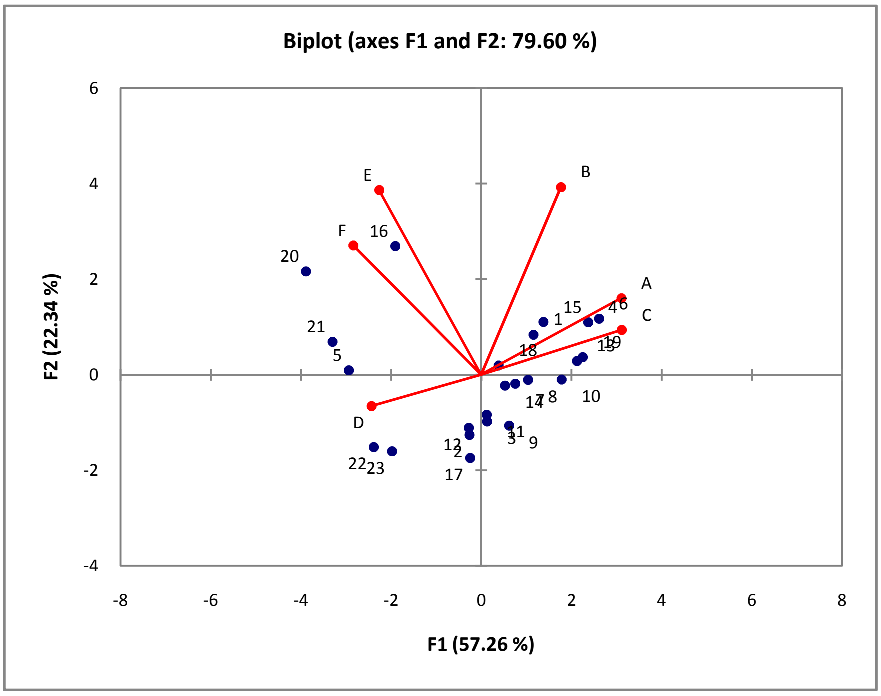

Tab. S1. Principal component analysis. Eigenvectors and correlation between variables and first two principal components (PC1,2) for behavioral measures:(A) time spent near the mirror, (B) the number of restrained attack, (C) the number of overt attack, (D) shuttled frequencies, (E) $CL_{16\text{-}30min\text{–}Abs}$, (F) $RT_{16\text{-}30min\ \text{-}Abs}$. See the methods section for details.

Principal component	%variance		A	B	C	D	E	F
PC1	57.26	Eigenvectors	0.482	0.274	0.483	-0.378	-0.351	-0.440
		correlation	0.893	0.508	0.895	-0.700	-0.651	-0.816
PC2	22.34	Eigenvectors	0.249	0.610	0.145	-0.101	0.599	0.419
		correlation	0.288	0.706	0.168	-0.117	0.694	0.486

Video 1. Proactive individual was tested for the aggressive behavioral experiment. Fish were tracked within 1cm near the mirror. Mirror was placed at the bottom of the screen.

Video 2. Reactive individual was tested for the aggressive behavioral experiment. Fish were tracked within 1cm near the mirror. Mirror was placed at the bottom of the screen.

Video 3. Swimming behavior of proactive individual performed before salinity stress.

Video 4. Swimming behavior of proactive individual performed during salinity stress (15ppt).

Video 5. Swimming behavior of reactive individual performed before salinity stress.

Video 6. Swimming behavior of reactive individual performed during salinity stress (15ppt).
